# Effect of MEF2A and SLC22A3-LPAL2-LPA gene polymorphisms on warfarin sensitivity and responsiveness in Jordanian cardiovascular patients

**DOI:** 10.1371/journal.pone.0294226

**Published:** 2023-11-10

**Authors:** Laith N. AL-Eitan, Ayah Y. Almasri, Adan H. Alnaamneh, Ahmad Mihyar

**Affiliations:** Department of Biotechnology and Genetic Engineering, Jordan University of Science and Technology, Irbid, Jordan; University of Cincinnati, INDIA

## Abstract

**Aims:**

This study aims to investigate the influence of *MEF2A* and *SLC22A3-LPAL2-LPA* polymorphisms on cardiovascular disease susceptibility and responsiveness to warfarin medication in Jordanian patients, during the initiation and maintenance phases of treatment.

**Backgrounds:**

Several candidate genes have been reported to be involved in warfarin metabolism and studying such genes may help in finding an accurate way to determine the needed warfarin dose to lower the risk of adverse drug effects, resulting in more safe anticoagulant therapy.

**Methods:**

The study population included 212 cardiovascular patients and 213 healthy controls. Genotyping of *MEF2A* and *SLC22A3-LPAL2-LPA* polymorphisms was conducted to examine their effects on warfarin efficiency and cardiovascular disease susceptibility using PCR-based methods.

**Results:**

One SNP (SLC22A3-LPAL2-LPA rs10455872) has been associated with cardiovascular disease in the Jordanian population, whereas the other SNPs in the MEF2A gene and SLC22A3-LPAL2-LPA gene cluster did not have any significant differences between cardiovascular patients and healthy individuals. Moreover, SLC22A3-LPAL2-LPA rs10455872 was correlated with moderate warfarin sensitivity, the other SNPs examined in the current study have not shown any significant associations with warfarin sensitivity and responsiveness.

**Conclusion:**

Our data refer to a lack of correlation between the MEF2A polymorphism and the efficacy of warfarin treatment in both phases of treatment, the initiation, and maintenance phases. However, only rs10455872 SNP was associated with sensitivity to warfarin during the initiation phase. Furthermore, rs3125050 has been found to be associated with the international normalized number treatment outcomes in the maintenance phase.

## Introduction

Anticoagulant therapy has increasingly been used in the treatment and prevention of several cardiovascular diseases and thrombotic disorders [1, 2], where these anticoagulant drugs work by keeping the international normalized ratio (INR) within the target range. INR, a measure of thrombotic status, must remain within the therapeutic range to prevent any thrombotic complications, where the lower INR is related to an increased risk of thrombosis or stroke, while the higher INR is related to an increased risk of bleeding [3].

Warfarin (3-(α-acetonylbenzyl)-4-hydroxycoumarin) is the most well-known anticoagulant drug that has been prescribed for therapeutic purposes for six decades for its efficacy in the treatment and prevention of several thrombotic disorders such as thrombosis, atrial fibrillation and after orthopaedic surgery [3, 4]. Warfarin work by deactivating the vitamin K epoxide reductase C complex (VKORC1), therefore, inhibiting the reproduction of vitamin K reduced form, which decreases the activity of coagulation factors II, VII, IX, and X [5]. Warfarin efficacy and safety mainly depend on the maintenance of the INR within the target range. However, the warfarin dosage is determined by a trial-and-error approach until reaching the appropriate dose and the target INR, which increases the risk of adverse drug effects at the beginning stage of treatment [6, 7], where a lower or higher dosage than demanded could cause to thrombotic risk and bleeding, respectively [8]. The wide inter-individual variability in warfarin responsiveness may be explained due to age, gender, smoking status, body mass index, dietary vitamin K content, concomitant diseases such as impaired liver function, and heart failure, and the use of concomitant medications [9–12], in addition to inter-individual genetic variation [13, 14].

Several studies have found that there are at least 30 genes that modulate warfarin response and sensitivity [15]. Genetic variants of the *VKORC1* and *CYP2C9* genes were considered the most important polymorphisms that correlated with variability in warfarin dose and the time required to obtain the target INR [16–18]. However, several genes have been found to affect different cardiovascular disorders, such as the *SLC22A3-LPAL2-LPA* gene cluster [19], and some of these genes considered disease-causing genes such as the myocyte enhancer factor 2A (*MEF2A*) gene [20], a member of the MEF2 family, together with MEF2B, MEF2C, and MEF2D encodes the transcription factors that control the expression of numerous muscle-specific genes. However, in several studies, polymorphisms within this gene have been linked to Coronary Artery Disease (CAD) and Myocardial Infarction (MI) [21, 22]. *SLC22A3-LPAL2-LPA* gene cluster consists of three genes: solute carrier family 22 member 3 (*SLC22A3*) gene, lipoprotein(a)-like 2 (*LPAL2*) gene, and lipoprotein(a) *(LPA*) gene, located on chromosome 6q26-27 [23]. This gene cluster was identified by Tre´gouet et al. as a significant risk factor locus for CAD by a genome-wide haplotype association (GWHA) study [19, 24]. The *LPA* gene within this cluster expresses the apolipoprotein(a) segment of the Lp(a) lipoprotein particle, which make this cluster involved with cardiovascular diseases by impacting the plasma lipid levels. *SLC22A3* gene that encodes organic cation transporter 3 (OCT3), playing a significant role in the transporting and clearance of toxic substances of many drugs, including metformin and histamine [25, 26], and several studies confirmed an association between *SLC22A3* polymorphisms and the increased susceptibility to cardiovascular diseases [27, 28]. *MEF2A* and *SLC22A3-LPAL2-LPA* have been studied widely in their correlation with cardiovascular diseases in multiple ethnicities and have been both identified as risk factors for cardiovascular diseases [19, 21, 29, 30]. Genetic screening for multiple polymorphisms in Jordanian cardiovascular disease patients has been studied for multiple genes that are reported with warfarin responsiveness [31–34], however there is no record of studies that corelate *MEF2A* and *SLC22A3-LPAL2-LPA* with warfarin responsiveness in Jordanian cardiovascular disease patients. Increasing candidate genes that undergo warfarin pharmacodynamics and pharmacokinetics and studying their impacts on warfarin efficacy may help to find a precise way to adjust the appropriate dose of warfarin and reduce the risk of adverse drug reactions, resulting in more safe anticoagulant therapy. Therefore, the objective of this study is to investigate the impacts of the *MEF2A* and *SLC22A3-LPAL2-LPA* SNPs on warfarin treatment efficacy during the initiation and maintenance phases of treatment in Jordanian cardiovascular patients for the first time.

## Materials and methods

### Study design and subjects

The Human Ethics Committees of the Jordan University of Science and Technology and the Royal Medical Service in Jordan have confirmed this study Confirmation code: 13/78/2014. After providing information on the purpose of the study and giving written informed consent from both healthy and patient blood donors, 212 cardiovascular patients were selected from the Queen Alia Heart Institute (QAHI) (anticoagulation clinic) in Amman, Jordan, and 213 healthy control subjects characterized by being between 18 to 85 years old and did not have heart disease were enrolled in this study. All subjects fitted the following inclusion criteria; the participants were (50% female and 50% male) 18-year-old or older patients who have been receiving warfarin as an anticoagulant drug for at least three months and have regularly attended the anticoagulant clinic. While patients who had lack medical data or did not provide their written consent, alcoholics, pregnant women, or taking any warfarin-interacting medications were excluded from the study. These interacting medications have been determined based on Dutch standards for the treatment of coumarin interactions [35].

Firstly, 350 cardiovascular patients were analyzed, and 138 were excluded from the study due to the inclusion and exclusion criteria, incomplete treatment plan, and failure of genotyping as described by AL-Eitan et al. [34]. Only 139 cardiovascular patients from the remaining 212 patients achieved the maintenance phase of treatment.

### Data collection and follow-up time

All medical and demographic data were gathered during the patient’s clinical visits. as described by Al-Eitan et al. [36].

### SNP selection and genotyping

*MEF2A* gene codes for a transcription factor that is solely expressed in coronary atrial endothelial cells. Reports have corelated a 7 amino acid deletion mutation in exon11 this transcription factor with coronary artery diseases along with other missense mutations in this gene [30]. The *SLC22A3-LPAL2-LPA* gene cluster majorly contributes to the concentrations of Lipoprotein (a) in the body, which has been identified as a risk factor for developing coronary artery diseases [37]. The *LPA* gene in the cluster is responsible for the production of the apolipoprotein (a) component in the lipoprotein [38]. Multiple reports have corelated SNPs in the gene cluster and high lipoprotein (a) concentration with risk of cardiovascular diseases [38, 39]. Therefore, a total of 11 SNPs in *MEF2A* and *SLC22A3-LPAL2-LPA* genes were selected from public databases like National Center for Biotechnology Information (NCBI) (http://www.ncbi.nlm. nih.gov/SNP) and the Applied Biosystems SNP database (http://www.appliedbiosystems). More information about the polymorphisms is listed in **(S1 Table in S1 File)**. DNA was extracted from the blood samples using the commercially available Wizard Genomic DNA Purification Kit (Promega) according to the manufacturer’s instructions. Genotyping was carried out at the Australian Genome Research Facility (AGRF) by using the Mass ARRAY system (iPLEX GOLD). The primers information is available in **(S2 Table in S1 File)** used for the *MEF2A*, and *SLC22A3-LPAL2-LPA* genes and the Mass ARRAYTM system outcomes are available in **(S1-S3 Figs in S1 File)** [32].

### Outcome measure

The warfarin dosage and INR values were collected from the patient’s medical records, in two different phases, the first was at the beginning of therapy where the warfarin dose and INR values are unstable, and this phase is called the initial phase of treatment. In this phase, all the patients follow the Royal Medical Services (RMS) Anticoagulation protocol by starting their warfarin treatment with 2.5 to 10mg doses taken at night on a daily bases. Older patients or patients with higher INR could be subject to lower doses as per the protocol. The second phase is called the maintenance phase of treatment, in which the warfarin dose is determined to be the mean value of the weekly dose at which the patient’s INR is stable and remains within the therapeutic range for two consecutive visits.

### Warfarin sensitivity and responsiveness measure

Both sensitivity and responsiveness were monitored throughout the study by monitoring the warfarin daily intake dose prescribed that would keep the INR value within the borders the treatment range. After the initiation of the treatment, the patients’ medical record were used along with personal interviews to identify the dose of warfarin the patients take to maintain their INR values.

Patients in this study were classified into three groups based on warfarin sensitivity depending on Gordon’s (2009) study [40]:

1. Warfarin resistance or poor metabolizer group, where the high daily dose of warfarin is needed to maintain the INR value within the treatment range (warfarin required dose> 49 mg/week).
2. Warfarin response or moderate metabolizer group, where moderate warfarin doses were needed (Warfarin required dose between 21 and 49 mg/week).
3. Warfarin sensitive or extensive metabolizer group, where the low warfarin doses were needed. (Warfarin required dose < 21 mg/week).

Furthermore, patients were classified into three groups based on warfarin responsiveness depending on Higashi et al. (2002) study [41]:

1. Good responders’ group, with INR values among the therapeutic range.
2. Poor responders’ group, with INR values below the therapeutic range.
3. Extensive responders’ group, with INR values over the therapeutic range.

### Statistical analysis

Microsoft Excel was used to calculate discrepancy and call rates, while the Pearson *χ*2 test was applied to estimate the aberration from Hardy Weinberg Equilibrium (HWE). For the genetic distribution, the Minor Allele Frequencies (MAF) and the Hardy-Weinberg equilibrium -p values were obtained using the Court lab-HW calculator. To find out which of the candidate SNPs is related to warfarin sensitivity and responsiveness, several statistical analyses were conducted, including the Chi-square test, unidirectional analysis of variance, and Turkey HSD post hoc multiple comparison tests. SPSS version 21.0 was utilized to conduct all analyses.

### Multiple testing corrections

The effective number of SNPs was evaluated according to the published literature [35], Moreover, Bonferroni correction was conducted at α/n where α = 0.05 and n represents the number of SNPs [36].

## Results

### Study population

The study group consists of 212 warfarin intake patients and was used to evaluate the effect of *MEF2A* and *SLC22A3-LPAL2-LPA* SNPs on the warfarin efficiency during the initiation phase of treatment. The age of the participants ranged between 18 to 85, mean age (±SD) of 56.03±17.68. A total of 139 patients achieved the maintenance phase. Therefore, they were used to evaluate the effect of these polymorphisms on warfarin treatment during the maintenance phase.

Overall, there were 25 extensive responders (18.0%), 88 moderate responders (63.3%), and 26 poor responders (18.7%). The clinical and demographic characteristics of each of the three groups were collected and summarized in a previous study by Al-Eitan et al. [34]. All 7 SNPs examined in this study, except rs2048327 SNP of the *SLC22A3-LPAL2-LPA* gene cluster were following with the HWE. All the 7 SNPs were polymorphic and therefore non were excluded from the study. The 7 polymorphic SNPs have passed quality-control tests with high accuracy and a low degree of discrepancy. The minor alleles and their frequencies for the successful genotyped SNPs are shown in **(S3 Table in S1 File)**.

### Polymorphism frequencies of *MEF2A* and *SLC22A3-LPAL2-LPA*genes and susceptibility between patients and control

Genotypic and allelic frequencies of *MEF2A* and *SLC22A3-LPAL2-LPA* genes in cardiovascular patients and healthy controls are shown in Table 1. Only rs10755578 SNP of the *SLC22A3-LPAL2-LPA* gene cluster ((CC+CG)/GG) was different significantly among patients and healthy controls (*P* = 0.04). Moreover, no significant associations were found between *MEF2A* and *SLC22A3-LPAL2-LPA* haplotypes and cardiovascular disease among patients and healthy controls (*P* ˃ 0.05) **(S4 Table in S1 File)**.

**Table 1:** The distributions of *MEF2A* and *SLC22A3-LPAL2-LPA* SNPs in 212 cardiovascular patients and 213 healthy controls.

Gene	SNP ID	Model	Patients %	Controls %	*p*-value*
***MEF2A***	rs8036677	GG/GA/AA	77.2/21.3/1.4	80.8/18.3/0.9	0.65
		GG/(GA+AA)	77.2/22.8	80.8/19.2	0.38
		(GG+GA)/AA	98.6/1.4	99.1/0.9	0.64
***SLC22A3-LPAL2-LPA***	rs10455872	AA/AG	97.2/2.8	98.6/1.4	0.3
	rs10755578	CC/CG/GG	41.8/46.1/12	34.1/46.5/19.4	0.07
		CC/(CG+GG)	41.8/58.2	34.1/65.9	0.1
		(CC+CG)/GG	88/12	80.6/19.4	0.04
	rs2048327	TT/CT/CC	64.2/27.8/8	67.1/26.8/6.1	0.6
		TT/(CT+CC)	64.2/35.8	67.1/32.9	0.52
		(TT+CT)/CC	92/8	93.9/6.1	0.44
	rs3125050	AA/CA	84/14	82.6/17.4	0.71
	rs3127583	GG/AG/AA	82.5/17.5/0.0	81.2/18.3/0.5	0.48
		GG/(AG+AA)	82.5/17.5	81.2/18.8	0.72
		(GG+AG)/AA	100/0.0	99.5/0.5	0.24
	rs3127599	CC/CT/TT	59.4/35.5/5.1	53.4/36.8/9.8	0.15
		CC/(CT+TT)	59.4/40.6	53.4/46.6	0.23
		(CC+CT)/TT	94.9/5.1	90.2/9.8	0.07
	rs366920	CC/TC/TT	33/44.8/22.2	32.4/47.9/19.7	0.77
		CC/(TC+TT)	33/67	32.4/67.6	0.89
		(CC+TC)/TT	77.8/22.2	80.3/19.7	0.53
	rs7767084	TT/CT/CC	73.1/23.1/3.8	69/26.8/4.2	0.65
		TT/(CT+CC)	73.1/26.9	69/31	0.35
		(TT+CT)/CC	96.2/3.8	95.5/4.2	0.81

*Chi–Square Test with p<0.05 is considered significant. Multiple statistical comparisons with p<0.005 is considered significant.

### Association of *MEF2A* and *SLC22A3-LPAL2-LPA* polymorphisms and warfarin sensitivity

Regarding the relationship between *MEF2A* and *SLC22A3-LPAL2-LPA* polymorphisms and warfarin sensitivity among the three inclusion groups (sensitive, moderate, and resistant), all SNPs showed no significant diversity among the warfarin-sensitive groups at the initiation phase of treatment (*P* ˃ 0.05) (**Table 2**). During the maintenance phase, a significant association was found between rs10455872 SNP of *SLC22A3-LPAL2-LPA* gene cluster and warfarin sensitivity (*P* = 0.02) (**Table 3**). Carriers of rs10455872 SNP (AA and GA) had a moderate sensitivity to warfarin. Furthermore, no significant correlation was remarked between *MEF2A* and *SLC22A3-LPAL2-LPA* haplotypes and sensitivity to warfarin (*P* ˃ 0.05) **(S5 Table in S1 File)**.

**Table 2:** Association of *MEF2A* and *SLC22A3-LPAL2-LPA* polymorphisms with warfarin sensitivity during the initiation phase of therapy of 212 warfarin-treated cardiovascular patients.

Gene	SNP ID	Genotype	Sensitive^a^	Moderate^b^	Resistance^c^	Overall *P*-value*
***MEF2A***	rs8036677	AA	(0/3) 0.0%	(2/3) 66.7%	(1/3) 33.3%	0.96
		**P-value** *	0.76	1	0.99	
		GA	(7/45) 15.6%	(32/45) 71.1%	(6/45) 13.3%	
		**P-value** *	1	0.93	0.94	
		GG	(25/163) 15.3%	(111/163) 68.1%	(27/163) 16.6%	
		**P-value** *	0.72	0.85	0.95	
***SLC22A3-LPAL2-LPA***	rs10455872	AA	(31/206) 15.0%	(144/206) 70.0%	(31/206) 15.0%	0.1
		**P-value** *	1	0.16	0.07	
		GA	(1/6) 16.7%	(2/6) 33.3%	(3/6) 50%	
		**P-value** *	1	0.16	0.07	
	rs10755578	CC	(13/87) 15.0%	(57/87) 65.5%	(17/87) 19.5%	0.48
		**P-value** *	1	0.62	0.47	
		GC	(16/96) 16.7%	(69/96) 71.8%	(11/96) 11.5%	
		**P-value** *	0.8	0.74	0.27	
		GG	(2/25) 8.0%	(18/25) 72.0%	(5/25) 20.0%	
		**P-value** *	0.59	0.95	0.84	
	rs2048327	CC	(1/17) 5.9%	(13/17) 76.5%	(3/17) 17.6%	0.79
		**P-value** *	0.54	0.78	0.98	
		CT	(11/59) 18.6%	(39/59) 66.1%	(9/59) 15.3%	
		**P-value** *	0.67	0.87	0.98	
		TT	(20/136) 14.7%	(94/136) 69.1%	(22/136) 16.2%	
		**P-value** *	0.98	1	1	
	rs3125050	AA	(29/178) 16.3%	(122/178) 68.5%	(27/178) 15.2%	0.45
		**P-value** *	0.54	0.97	0.73	
		CA	(3/34) 8.8%	(24/34) 70.6%	(7/34) 20.6%	
		**P-value** *	0.54	0.97	0.73	
	rs3127599	CC	(17/117) 14.5%	(81/117) 69.2%	(19/117) 16.2%	0.65
		**P-value** *	0.95	0.97	1	
		CT	(13/70) 18.6%	(46/70) 65.7%	(11/70) 15.7%	
		**P-value** *	0.63	0.82	0.99	
		TT	(0/10) 0.0%	(8/10) 80.0%	(2/10) 20.0%	
		**P-value** *	0.39	0.73	0.95	
	rs7767084	CC	(2/8) 25.0%	(6/8) 75.0%	(0/8) 0.0%	0.58
		**P-value** *	0.73	0.93	0.45	
		CT	(8/49) 16.3%	(35/49) 71.4%	(6/49) 12.2%	
		**P-value** *	0.96	0.91	0.71	
		TT	(22/155) 14.2%	(105/155) 67.7%	(28/155) 18.1%	
		**P-value** *	0.84	0.84	0.41	

* Chi-square test with p value < 0.05 is considered significant. Multiple statistical comparisons with p<0.005 is considered significant.

^a.^ Warfarin Sensitive group (required minimum dose < 21 mg/week).

^b.^ Warfarin Intermediate response group (required average dose between 21 and 49 mg/week).

^c.^ Warfarin Resistance group (required high dose > 49 mg/week).

**Table 3:** Association of *MEF2A* and *SLC22A3-LPAL2-LPA* polymorphisms with warfarin sensitivity during the maintenance phase of therapy of 139 warfarin-treated cardiovascular patients.

Gene	SNP ID	Genotype	Sensitive^a^	Moderate^b^	Resistance^c^	Overall *P*-value*
***MEF2A***	rs8036677	AA	(0/3) 0.0%	(1/3) 33.3%	(2/3) 66.7%	0.13
		**P-value** *	0.77	0.59	0.21	
		GA	(6/31) 19.4%	(22/31) 71.0%	(3/31) 9.7%	
		**P-value** *	0.68	0.47	0.11	
		GG	(14/104) 13.5%	(62/104) 59.6%	(28/104) 26.9%	
		**P-value** *	0.84	0.70	0.35	
***SLC22A3-LPAL2-LPA***	rs10455872	AA	(18/134) 13.4%	(86/134) 64.2%	(30/134) 22.4%	0.02
		**P-value** *	0.25	0.01	0.15	
		GA	(2/5) 40.0%	(0/5) 0.0%	(3/5) 60.0%	
		**P-value** *	0.25	0.01	0.15	
	rs10755578	CC	(9/61) 14.8%	(37/61) 60.7%	(15/61) 24.6%	0.97
		**P-value** *	1	0.97	0.97	
		GC	(9/57) 15.8%	(36/57) 63.2%	(12/57) 21.0%	
		**P-value** *	0.96	0.96	0.85	
		GG	(2/18) 11.1%	(11/18) 61.1%	(5/18) 27.8%	
		**P-value** *	0.90	1	0.90	
	rs2048327	CC	(3/9) 33.3%	(4/9) 44.4%	(2/9) 22.2%	0.38
		**P-value** *	0.25	0.54	1	
		CT	(4/41) 9.8%	(25/41) 61.0%	(12/41) 29.3%	
		**P-value** *	0.60	0.99	0.61	
		TT	(13/89) 14.6%	(57/89) 64.0%	(19/89) 21.3%	
		**P-value** *	1	0.78	0.68	
	rs3125050	AA	(17/117) 14.5%	(73/117) 62.4%	(27/117) 23.1%	0.91
		**P-value** *	1	0.96	0.91	
		CA	(3/22) 13.6%	(13/22) 59.1%	(6/22) 27.3%	
		**P-value** *	1	0.96	0.91	
	rs3127599	CC	(9/77) 11.7%	(50/77) 64.9%	(18/77) 23.4%	0.77
		**P-value** *	0.61	0.47	0.84	
		CT	(8/44) 18.2%	(24/44) 54.5%	(12/44) 27.3%	
		**P-value** *	0.64	0.59	0.93	
		TT	(1/6) 16.7%	(3/6) 50.0%	(2/6) 33.3%	
		**P-value** *	0.99	0.86	0.90	
	rs7767084	CC	(1/6) 16.7%	(5/6) 83.3%	(0/6) 0.0%	0.56
		**P-value** *	0.99	0.54	0.38	
		CT	(3/31) 9.7%	(21/31) 67.7%	(7/31) 22.6%	
		**P-value** *	0.70	0.75	0.99	
		TT	(16/102) 15.7%	(60/102) 58.8%	(26/102) 25.5%	
		**P-value** *	0.77	0.47	0.72	

*Chi-square test with p-value < 0.05 is considered significant. Multiple statistical comparisons with p<0.005 is considered significant.

^a.^ Warfarin Sensitive group (required minimum dose < 21 mg/week).

^b.^ Warfarin Intermediate response group (required average dose between 21 and 49 mg/week).

^c.^Warfarin Resistance group (required high dose > 49 mg/week).

In the case of the association between *MEF2A* and *SLC22A3-LPAL2-LPA* SNPs and warfarin required dose, no significant correlation was noticed during the initiation and maintenance phases of treatment (*P* ˃ 0.05) (**Table 4**). The post-hock multiple comparisons test was conducted to test the significance of the warfarin required dose and *MEF2A* and *SLC22A3-LPAL2-LPA* SNPs. Moreover, the post-hock test revealed that there was no significant correlation between the studied SNPs and the warfarin required dose (*P* ˃ 0.05) **(S6 Table in S1 File)**.

**Table 4:** Association of *MEF2A* and *SLC22A3-LPAL2-LPA* polymorphisms with variability on warfarin required doses.

Gene	SNP ID	Genotype	Initiation dose	Overall *P*-value*	Maintenance dose	Overall *P*-value*
***MEF2A***	rs8036677	AA	50.83 [3.35]	0.48	48.50 [3.21]	0.54
		GA	35.68 [12.0]		36.88 [18.81]	
		GG	38.57 [25.45]		38.93 [17.84]	
***SLC22A3-LPAL2-LPA***	rs10455872	AA	37.97 [23.20]	0.74	38.36 [17.71]	0.50
		GA	41.13 [19.48]		43.86 [23.47]	
	rs10755578	CC	40.06 [31.35]	0.42	37.94 [17.25]	0.70
		GC	35.90 [13.28]		38.24 [17.67]	
		GG	40.52 [19.36]		41.90 [21.51]	
	rs2048327	CC	38.28 [14.38]	0.81	37.32 [22.40]	0.42
		CT	36.38 [13.81]		41.66 [18.57]	
		TT	38.76 [26.91]		37.25 [17.09]	
	rs3125050	AA	37.81 [24.45]	0.72	38.36 [18.06]	0.77
		CA	39.37 [13.98]		39.60 [17.18]	
	rs3127599	CC	39.08 [27.83]	0.47	39.31 [28.50]	0.54
		CT	35.80 [14.20]		37.74 [16.05]	
		TT	44.18 [25.09]		46.63 [36.6]	
	rs7767084	CC	29.43 [9.69]	0.39	27.75 [8.17]	0.24
		CT	35.91 [13.42]		41.17 [19.18]	
		TT	39.18 [25.74]		38.40 [17.74]	

* One-way ANOVA test with *P*value < 0.05 is considered significant, Mean Standard deviation in square brackets. Multiple statistical comparisons with p<0.005 is considered significant.

### Association of *MEF2A* and *SLC22A3-LPAL2-LPA* polymorphisms and warfarin responsiveness

Based on the association of *MEF2A* and *SLC22A3-LPAL2-LPA* polymorphisms and warfarin responsiveness among the three warfarin response groups (poor, good, and extensive responders), there were no significant differences between *MEF2A* and *SLC22A3-LPAL2-LPA* polymorphisms either in the initiation phase or maintenance phase of therapy (*P* ˃ 0.05) (**Tables 5 and 6**). However, a significant association was found between ACCAGCTC *SLC22A3-LPAL2-LPA* haplotype and responsiveness to warfarin (*P* = 0.005) **(S7 Table in S1 File)**. Among all warfarin intake patients, no significant differences were found regarding *MEF2A* and *SLC22A3-LPAL2-LPA* polymorphisms and initial INR values (*P* ˃ 0.05) **(S8 Table in S1 File)**. In contrast, a significant difference was observed between rs3125050 *SLC22A3-LPAL2-LPA* SNP and the INR values measured during the maintenance phase of treatment (*P* = 0.02; **Table 7**).

**Table 5:** Association of *MEF2A* and *SLC22A3-LPAL2-LPA* polymorphisms with warfarin responsiveness during the initiation phase of therapy of 212 warfarin-treated cardiovascular patients.

Gene	SNP ID	Genotype	Poor Responder	good Responder	Extensive Responder	Overall p-value*
***MEF2A***	rs8036677	AA	(0/3) 0.0%	(3/3) 100%	(0/3) 0.0%	0.44
		**P-value** *	0.71	0.6	0.48	
		GA	(6/45) 13.3%	(38/45) 84.4%	(1/45) 2.2%	
		**P-value** *	0.62	0.35	0.24	
		GG	(33/163) 20.2%	(120/163) 73.6%	(10/163) 6.1%	
		**P-value** *	0.92	0.59	0.54	
***SLC22A3-LPAL2-LPA***	rs10455872	AA	(38/206) 18.4%	(157/206) 76.2%	(11/206) 5.3%	1
		**P-value** *	1	0.92	0.84	
		GA	(1/6) 16.7%	(5/6) 83.3%	(0/6) 0.0%	
		**P-value** *	1	0.92	0.84	
	rs10755578	CC	(17/87) 19.5%	(64/87) 73.6%	(6/87) 6.9%	0.48
		**P-value** *	1	0.62	0.47	
		GC	(18/96) 18.8%	(73/96) 76.0%	(5/96) 5.2%	
		**P-value** *	0.8	0.74	0.27	
		GG	(3/25) 12.0%	(22/25) 88.0%	(0/25) 0.0%	
		**P-value** *	0.59	0.95	0.84	
	rs2048327	CC	(2/17) 11.8%	(14/17) 82.4%	(1/17) 5.9%	0.7
		**P-value** *	0.76	0.84	0.99	
		CT	(8/59) 13.6%	(48/59) 81.4%	(3/59) 5.1%	
		**P-value** *	0.53	0.57	1	
		TT	(29/136) 21.3%	(100/136) 73.5%	(7/136) 5.1%	
		**P-value** *	0.34	0.42	1	
	rs3125050	AA	(35/178) 19.7%	(133/178) 74.7%	(10/178) 5.6%	0.41
		**P-value** *	0.55	0.41	0.81	
		CA	(4/34) 11.8%	(29/34) 85.3%	(1/34) 2.9%	
		**P-value** *	0.55	0.41	0.81	
	rs3127599	CC	(20/117) 17.1%	(90/117) 76.9%	(7/117) 6.0%	0.3
		**P-value** *	0.76	0.95	0.78	
		CT	(17/70) 24.3%	(51/70) 72.9%	(2/70) 2.9%	
		**P-value** *	0.34	0.72	0.57	
		TT	(0/10) 0.0%	(9/10) 90.0%	(1/10) 10.0%	
		**P-value** *	0.3	0.57	0.77	
	rs7767084	CC	(1/8) 12.5%	(7/8) 87.5%	(0/8) 0.0%	0.82
		**P-value** *	0.91	0.75	0.8	
		CT	(7/49) 14.3%	(39/49) 79.6%	(3/49) 6.1%	
		**P-value** *	0.7	0.84	0.95	
		TT	(31/155) 20.0%	(116/155) 74.8%	(8/155) 5.2%	
		**P-value** *	0.61	0.67	1	

*Chi-square test with p value < 0.05 is considered significant. Multiple statistical comparisons with p<0.005 is considered significant.

**Table 6:** Association of *MEF2A* and *SLC22A3-LPAL2-LPA* SNPs with warfarin responsiveness during the maintenance phase of therapy of 139 warfarin-treated cardiovascular patients.

Gene	SNP ID	Genotype	Poor Responder	good Responder	Extensive Responder	Overall p-value*
***MEF2A***	rs8036677	AA	(0/3) 0.0%	(3/3) 100.0%	(0/3) 0.0%	0.85
		**P-value** *	0.90	0.83	0.93	
		GA	(1/31) 3.2%	(29/31) 93.5%	(1/31) 3.2%	
		**P-value** *	0.70	0.67	0.94	
		GG	(8/104) 7.7%	(91/104) 87.5%	(5/104) 4.8%	
		**P-value** *	0.62	0.56	0.90	
***SLC22A3-LPAL2-LPA***	rs10455872	AA	(9/134) 6.7%	(119/134) 88.8%	(6/134) 4.5%	0.73
		**P-value** *	0.84	0.73	0.89	
		GA	(0/5) 0.0%	(5/5) 100.0%	(0/5) 0.0%	
		**P-value** *	0.84	0.73	0.89	
	rs10755578	CC	(3/61) 4.9%	(54/61) 88.5%	(4/61) 6.6%	0.44
		**P-value** *	0.91	0.92	0.55	
		GC	(5/57) 8.8%	(50/57) 87.7%	(2/57) 3.5%	
		**P-value** *	0.48	0.81	0.91	
		GG	(0/18) 0.0%	(18/18) 100.0%	(0/18) 0.0%	
		**P-value** *	0.52	0.3	0.62	
	rs2048327	CC	(0/9) 0.0%	(8/9) 88.9%	(1/9) 11.1%	0.70
		**P-value** *	0.72	1	0.58	
		CT	(2/41) 4.9%	(37/41) 90.2%	(2/41) 4.9%	
		**P-value** *	0.89	0.97	0.98	
		TT	(7/89) 7.9%	(79/89) 88.8%	(3/89) 3.4%	
		**P-value** *	0.67	0.98	0.76	
	rs3125050	AA	(9/117) 7.7%	(102/117) 87.2%	(6/117) 5.1%	0.21
		**P-value** *	0.40	0.21	0.55	
		CA	(0/22) 0.0%	(22/22) 100.0%	(0/22) 0.0%	
		**P-value** *	0.40	0.21	0.55	
	rs3127599	CC	(4/77) 5.2%	(69/77) 89.6%	(4/77) 5.2%	0.57
		**P-value** *	0.59	0.96	0.66	
		CT	(5/44) 11.4%	(38/44) 86.4%	(1/44) 2.2%	
		**P-value** *	0.39	0.79	0.78	
		TT	(0/6) 0.0%	(6/6) 100.0%	(0/6) 0.0%	
		**P-value** *	0.70	0.68	0.88	
	rs7767084	CC	(0/6) 0.0%	(6/6) 100.0%	(0/6) 0.0%	0.89
		**P-value** *	0.81	0.68	0.87	
		CT	(2/31) 6.5%	(27/31) 87.0%	(2/31) 6.5%	
		**P-value** *	1	0.91	0.80	
		TT	(7/102) 6.9%	(91/102) 89.2%	(4/102) 3.9%	
		**P-value** *	0.89	1	0.93	

*Chi-square test with p value < 0.05 is considered significant. Multiple statistical comparisons with p<0.005 is considered significant.

**Table 7:** Association of *MEF2A* and *SLC22A3-LPAL2-LPA* polymorphisms with INR treatment outcome.

Gene	SNP ID	Genotype	Initiation INR	Overall P-value*	Maintenance INR	Overall P-value*
***MEF2A***	rs8036677	AA	2.67 [0.93]	0.88	2.50 [0.20]	0.32
		GA	2.47 [0.63]		2.78 [0.34]	
		GG	2.45 [0.81]		2.68 [0.40]	
***SLC22A3-LPAL2-LPA***	rs10455872	AA	2.47 [0.78]	0.16	2.70 [0.39]	0.67
		GA	2.02 [0.47]		2.62 [0.22]	
	rs10755578	CC	2.36 [0.72]	0.31	2.70 [0.38]	0.92
		GC	2.51 [0.83]		2.68 [0.41]	
		GG	2.56 [0.68]		2.72 [0.37]	
	rs2048327	CC	2.27 [0.60]	0.25	2.72 [0.26]	0.92
		CT	2.58 [0.87]		2.71 [0.38]	
		TT	2.43 [0.74]		2.68 [0.41]	
	rs3125050	AA	2.46 [0.76]	0.77	2.73 [0.40]	0.02
		CA	2.42 [0.82]		2.51 [0.24]	
	rs3127599	CC	2.42 [0.74]	0.14	2.71 [0.38]	0.67
		CT	2.42 [0.80]		2.65 [0.39]	
		TT	2.93 [1.09]		2.62 [0.58]	
	rs7767084	CC	2.58 [0.78]	0.70	2.78 [0.23]	0.84
		CT	2.52 [0.79]		2.70 [0.40]	
		TT	2.47 [0.77]		2.69 [0.40]	

* One-way ANOVA test with *P*value < 0.05 is considered significant, Mean Standard deviation in square brackets. Multiple statistical comparisons with p<0.005 is considered significant.

## Discussion

Warfarin is the most broadly used anticoagulant drug, despite it being complicated by high variability in dose among individuals, requiring regular clinic visits and repeating INR tests [5, 42]. Various genetic and environmental factors affect the dose prescribed and the individual response to warfarin [9, 13]. More than 30 genes were found to associate with warfarin efficiency [15]. Numerous studies have investigated the correlation between various gene families polymorphisms and variability in warfarin dose and time required to arrive at the therapeutic INR in Jordanians and other ethnicities [13, 14, 17, 34, 36, 43, 44]. However, there is no report stating the correlation between *MEF2A* and *SLC22A3-LPAL2-LPA* polymorphisms in Jordanian cardiovascular disease patients and warfarin responsiveness. Studying more genes may help in finding an accurate prediction of therapeutic response and safety before the prescription. Therefore, new genes (*MEF2A* and *SLC22A3-LPAL2-LPA*) were analyzed in this study to determine their effects on warfarin sensitivity and responsiveness.

*MEF2A* gene has a highly polymorphic genomic sequence, which added interest to be studied and analyzed [45]. In fact, various studies have confirmed the association of *MEF2A* polymorphisms with the autosomal dominant Coronary Artery Disease (adCAD1) and Myocardial Infarction (MI) [21, 46, 47], and the belief that approximately 2% of CAD is a result of mutations in the *MEF2A* gene [29], especially, the mutations within exon 11, where various insertion/deletion and substitution polymorphisms like a common variant (CAG)n polymorphism [48, 49]. On the other hand, various studies have identified *MEF2* polymorphisms in healthy people, therefore increasing the probability that this gene is not related to CAD [50]. Indeed, the association of *MEF2A* polymorphisms to CAD is controversial [50], Kajimoto et al. (2005) found that the *MEF2A* polymorphism is not contributed substantially to MI among the Japanese population [51], and Hsu et al. (2010) revealed that other Polymorphism in *MEF2A* is not linked to the Risk of CADin Taiwanese. Moreover, our study found that there is no significant association between *MEF2A*polymorphism and cardiovascular diseases among Jordanian patients (Table 1).

Several studies have determined the *SLC22A3-LPAL2-LPA* cluster as a powerful susceptibility region for CAD [19]. Where other studies found there is no association of *SLC22A3-LPAL2-LPA* polymorphisms with cardiovascular diseases. Ahani et al. found a lacking of rs10755578 SNP within this gene cluster with CAD in the Iranian population [52], and another study by Lv et al. Also found that there is no association between *SLC22A3-LPAL2-LPA* polymorphisms and CAD in the Chinese Han population [23]. The genotypic and allelic frequencies of the studied SNPs in this study did not significantly differ between patients and healthy subjects except for one SNP. We found rs10755578 SNP is associated with cardiovascular diseases in Jordanian patients (Table 1), supposedly because of its ability to control plasma lipid levels. This result is incompatible with Ahani et al. study, which may be due to the different lifestyles and other environmental factors between the two populations, in addition to possibly presenting other polymorphisms that impact the diseases.

Our study exhibited that the allelic frequencies of the *SLC22A3-LPAL2-LPA* polymorphisms were found to be drastically different from other populations [52, 53]. For instance, the frequency of wild genotype (CC) of rs10755578 SNP in the cardiovascular patients was 41.8% in our population and 29.0% in the Iranian population, and 46.5% was the frequency of heterozygous genotype (CG) in our population and 40.0% in Iranian population, and the frequency of the mutant genotype (GG) was 19.0% and 31.0% in Jordanian and Iranian, respectively [52].

To our knowledge, no studies illustrate the correlation between *MEF2A* and *SLC22A3-LPA L2-LPA* polymorphisms and warfarin efficiency. In the current study, we examine the relationship between *MEF2A* and *SLC22A3-LPAL2-LPA* SNPs and warfarin sensitivity and responsiveness throughout the initiation and maintenance phases of treatment in Jordanian cardiovascular patients. Our results strongly indicate that there is no significant correlation between *MEF2A* and *SLC22A3-LPAL2-LPA* polymorphisms and their haplotypes and warfarin sensitivity and the warfarin required doses during the initiation and maintenance phases of treatment. (*P* ˃ 0.05) (Tables 2–4). Only *SLC22A3-LPAL2-LPA* rs10455872 SNP exhibited a significant correlation with sensitivity to warfarin during the maintenance phase. Our results showed that this SNP is linked to moderate sensitivity of warfarin during the maintenance phase, where we found that 64.2% of moderate patients were homozygous (AA) for this SNP and 0.0% of them were heterozygous (GA), which revealed that there is a significantly different with total*P* = 0.02 (Table 3). The current study also showed that there is no significant relation between *MEF2A* and *SLC22A3-LPAL2-LPA* polymorphisms and responsiveness to warfarin during the initiation and maintenance phases of treatment (*P* ˃ 0.05) (Tables 5 and 6). There is only one haplotype block of the *SLC22A3-LPAL2-LPA* gene cluster (ACCAGCTC) revealed a significant relation with responsiveness to warfarin (*P* = 0.005). Besides, no significant differences were reported in our study regarding the INR measurements at the initiation phase of treatment with (*P* ˃ 0.05), On the other hand, there was a significant difference regarding the maintenance INR measurements with rs3125050 *SLC22A3-LPAL2-LPA* SNP with a (*P* = 0.02; Table 7).

## Conclusion

In the current study, we assessed the genotypic and allelic frequencies of the *MEF2A* and *SLC22A3-LPAL2-LPA* SNPs in the Jordanian population and then studied whether these polymorphisms influence the response and sensitivity to warfarin at the initiation and maintenance phases of treatment. We conclude that the carrying of *MEF2A* or *SLC22A3-LPAL2-LPA* SNPs is not linked to warfarin dose, sensitivity, or responsiveness, except *SLC22A3-LPAL2-LPA* rs10455872 SNP that impact warfarin sensitivity during the maintenance phase. Moreover, *SLC22A3-LPAL2-LPA* rs3125050 SNP is associated with the maintenance INR values during the maintenance phase. It is worth to note that there is a lack of studies in regards of the pharmacogenomic treatment options in the Arab region including Jordan [54, 55]. Thus, we recommend that this study must be verified in larger sample sizes and other populations; in order to find more accurate anticoagulant therapy based on personalized medicine.

## Supporting information

S1 File S1-S3 Figs and S1-S8 Tables. (DOCX)

S1 Data (XLSX)
