# Supplementary material for: Effect of MEF2A and SLC22A3-LPAL2-LPA gene polymorphisms on warfarin sensitivity and responsiveness in Jordanian cardiovascular patients
Source: PLoS One. 2023 Nov 10;18(11):e0294226. doi: 10.1371/journal.pone.0294226 (PMC10637663; doi:10.1371/journal.pone.0294226)
Supplement: S1 File — (DOCX) [file pone.0294226.s001.docx]

**Supplementary materials**

**
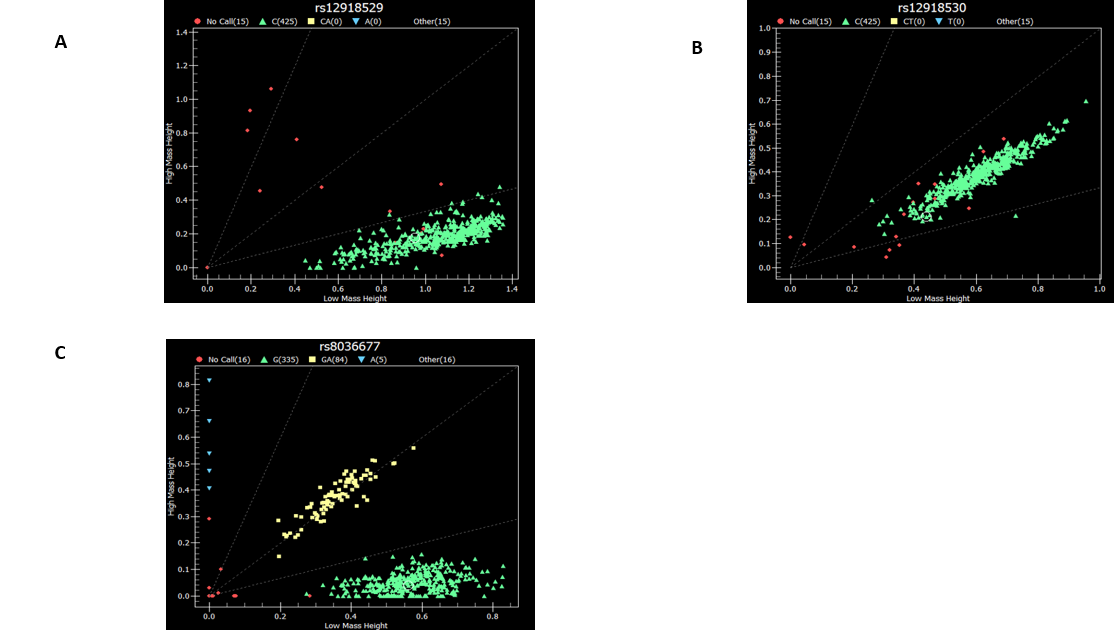
**

**Figure S1 Mass ARRAY analysis of *MEF2A* gene SNPs A)** rs12918529, **B)** rs12918530 and **C)** rs8036677.

**
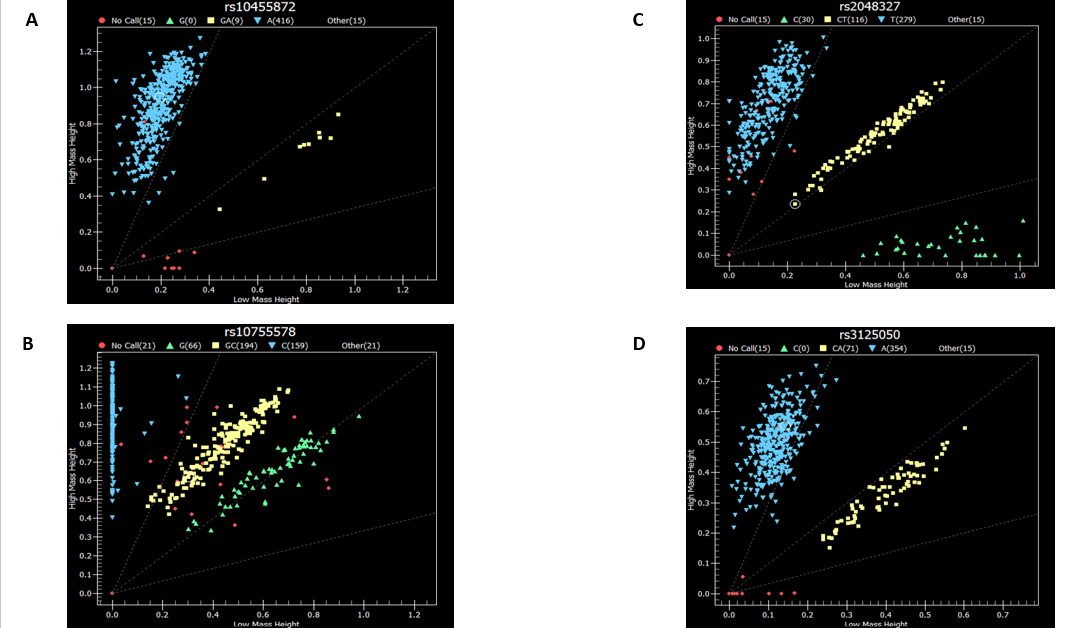
**

**Figure S2 Mass ARRAY analysis of *SLC22A3-LPAL2-LPA* gene SNPs: A)** rs10455872, **B)** rs10755578, **C)** rs2048327 and **D)** rs312050.

**Figure S3 Mass ARRAY analysis of *SLC22A3-LPAL2-LPA* gene SNPs: A)** rs3127583, **B)** rs3127599, **C)** rs366920 and **D)** rs7767084.

Table S1: Genes and SNPs’ characteristics

| **Gene** | **SNP ID** | **ChrPosition^a^** | **SNP** | **SNP Type** |
| --- | --- | --- | --- | --- |
| ***MEF2A*** | rs12918529 | 16:31349443 | C/A | N/A^b^ |
|  | rs12918530 | 16:2211713 | C/T | 3 Prime UTR Variant |
|  | rs8036677 | 15:99607837 | G/A | Intron Variant |
| ***SLC22A3-LPAL2-LPA*** | rs10455872 | 6:160589086 | A/G | Intron Variant |
|  | rs10755578 | 6:160548706 | C/G | Intron Variant |
|  | rs2048327 | 6:160442500 | T/C | Intron Variant |
|  | rs3125050 | 6:160319804 | A/C | N/A^b^ |
|  | rs3127583 | 6:160306099 | G/A | N/A^b^ |
|  | rs3127599 | 6:160486102 | C/T | Intron Variant |
|  | rs366920 | 6:160316918 | T/C | N/A^b^ |
|  | rs7767084 | 6:160541471 | T/C | Intron Variant |
| ^a.^ Chromosome positions are based on NCBI Human Genome Assembly Build.  ^b.^N/A: Not Avilable. | | | | |

**Table S2: Primer Information**

| **Gene** | **SNP** | **Primer 1** | **Primer 2** | **Extension Primer** |
| --- | --- | --- | --- | --- |
| ***MEF2A*** | rs12918529 | ACGTTGGATGCACCCACACATTTGCCCAT | ACGTTGGATGGTGGATAGGTTTGTGTGTGG | CCCATCCACCCTCCA |
|  | rs12918530 | ACGTTGGATGATCCACCTGCCTCAGCCTTC | ACGTTGGATGAGCAGAGCTCAGAAGTAATG | ccTGTGAGCCACTGCGCC |
|  | rs8036677 | ACGTTGGATGCTCCTTAGAGATAAGCATAG | ACGTTGGATGGGATGAAAGAGCATGGATGG | gaacGGAGACTTTGTAAACTGCC |
| ***SLC22A3-LPAL2-LPA*** | rs10455872 | ACGTTGGATGCACTTTCTCCTCTAACCTG | ACGTTGGATGGGCTGGCAACACATAGCTTT | cCCTCTAACCTGTATAAACACA |
|  | rs10755578 | ACGTTGGATGCAAAATGTAGAGGGTGCTGG | ACGTTGGATGGTAGTTCATTGTCAGGCCAC | GTGCTGGATGTCCCT |
|  | rs2048327 | ACGTTGGATGCAATGTTCCAATGTAGTGGC | ACGTTGGATGATGCCTTTCTGAGCAGGTTG | TGTAGTGGCTGCTCA |
|  | rs3125050 | ACGTTGGATGGCAATTGCTGTCAACCTCCT | ACGTTGGATGGCTGCCAACAAGGTTACTAC | ccacCCTCCTGATCATCTATCC |
|  | rs3127583 | ACGTTGGATGACAGAAGAGCCAACTGAACC | ACGTTGGATGGAATGGCCATAAAACCAGAC | gCATGATTTATCTTGGTTCCT |
|  | rs3127599 | ACGTTGGATGACCAGAAATGAGTCTTCAAG | ACGTTGGATGAGGAATCTGTTGCTTTTGTG | AATCATTCTCTTTTTTTTATTTGTAAG |
|  | rs366920 | ACGTTGGATGGCCTTGATTGGGCATTTAGC | ACGTTGGATGACAGAGCTGAGGATAAGACC | GGGCATTTAGCATTCTTTA |
|  | rs7767084 | ACGTTGGATGGAGCAAAGCTAAGTGACTCC | ACGTTGGATGTTGGGCTGGTCACTTTTGTC | AGAATGAAGCTCTATAGCTG |

| **Table S3: List of SNPs, their minor allele frequencies, and HWE p-values** | | | | | |
| --- | --- | --- | --- | --- | --- |
| **Gene** | **SNP ID** | **MA^a^** | **Patients MAF^b^** | **Controls MAF^b^** | **HWE^c^p-value** |
| ***MEF2A*** | rs8036677 | A | 0.12 | 0.1 | 1 |
| ***SLC22A3-LPAL2-LPA*** | rs10455872 | G | 0.01 | 0.01 | 1 |
|  | rs10755578 | G | 0.35 | 0.43 | 0.61 |
|  | rs2048327 | C | 0.22 | 0.19 | 0.001 |
|  | rs3125050 | C | 0.08 | 0.09 | 0.1 |
|  | rs3127583 | A | 0.09 | 0.1 | 0.24 |
|  | rs3127599 | T | 0.23 | 0.28 | 0.36 |
|  | rs366920 | T | 0.45 | 0.44 | 0.24 |
|  | rs7767084 | C | 0.15 | 0.18 | 0.08 |
| ^a.^ MA: Minor allele.  ^b.^ MAF: Minor allele frequency.  ^c.^ HWE: Hardy-Weinberg equilibrium. | | | | | |

| **Table S4: The distributions of *SLC22A3-LPAL2-LPA*haplotypes in 211 Cardiovascular patients compared to 213 healthy controls** | | | | | |
| --- | --- | --- | --- | --- | --- |
| **Gene** | **Haplotypes** | **Patients (%)** | **Controls (%)** | **Odds ratio (95% CI)** | **p-value^*^** |
| ***SLC22A3-LPAL2-LPA*** | ACTAGCCT | 84 (40%) | 85 (40%) | 1.00 | --- |
|  | AGTAGTTT | 21 (10%) | 28 (13%) | 0.68 (0.41 - 1.11) | 0.12 |
|  | ACTAGCTC | 21 (10%) | 19 (8%) | 1.26 (0.71 - 2.24) | 0.44 |
|  | AGCAGCTC | 17 (8%) | 19 (9%) | 0.86 (0.51 - 1.46) | 0.57 |
|  | AGTAGTCT | 15 (7%) | 19 (9%) | 0.76 (0.43 - 1.36) | 0.36 |
|  | AGCAGCCC | 6 (3%) | 9 (4%) | 0.57 (0.25 - 1.31) | 0.19 |
|  | ACTCACTT | 6 (3%) | 9 (4%) | 0.58 (0.23 - 1.45) | 0.24 |
|  | ACCAGCCT | 6 (3%) | 4 (2%) | 1.95 (0.71 - 5.37) | 0.2 |
|  | ACTAGTTT | 4 (2%) | 2 (1%) | 1.87 (0.57 - 6.14) | 0.3 |
|  | ACCAGCTT | 6 (3%) | 1 (0.4%) | 5.34 (0.89 - 31.87) | 0.07 |
|  | AGCCACTC | 2 (1%) | 2 (1%) | 0.76 (0.20 - 2.94) | 0.7 |
|  | AGTCATTT | 4 (2%) | 2 (1%) | 1.29 (0.27 - 6.26) | 0.75 |
|  | AGTAGCCC | 1 (0.3%) | 2 (2%) | 0.31 (0.04 - 2.13) | 0.23 |
| ^*^Chi–Square Test with p<0.05 is considered significant. | | | | | |

| **Table S5: The distributions of *SLC22A3-LPAL2-LPA*haplotypes among 212 warfarin sensitive patients** | | | | |
| --- | --- | --- | --- | --- |
| **Gene** | **Haplotypes** | **Frequency (%)** | **Odds ratio (95% CI)** | **p-value^*^** |
| ***SLC22A3-LPAL2-LPA*** | ACTAGCTC | 0.27 | 0.00 | --- |
|  | AGTAGCTC | 0.14 | 0.04 (-0.16 - 0.23) | 0.72 |
|  | ACTAGTTC | 0.13 | -0.06 (-0.26 - 0.14) | 0.54 |
|  | ACTAGCTT | 0.07 | -0.07 (-0.33 - 0.19) | 0.62 |
|  | AGCAGTCC | 0.04 | -0.22 (-0.51 - 0.08) | 0.16 |
|  | AGTAGTTC | 0.04 | -0.04 (-0.36 - 0.29) | 0.83 |
|  | AGTAGTTT | 0.04 | -0.29 (-0.63 - 0.04) | 0.09 |
|  | ACCAGCTC | 0.02 | 0.25 (-0.16 - 0.66) | 0.23 |
|  | ACCAGTCC | 0.02 | 0.08 (-0.3 - 0.47) | 0.67 |
|  | ACCAGTTC | 0.02 | -0.22 (-0.7 - 0.27) | 0.38 |
|  | ACTCATTC | 0.02 | 0.25 (-0.2 - 0.7) | 0.27 |
|  | AGTCATTT | 0.02 | 0.3 (-0.15 - 0.75) | 0.19 |
|  | ACTAGTCC | 0.02 | -0.32 (-0.77 - 0.13) | 0.17 |
|  | AGCAGTCT | 0.01 | 0.19 (-0.33 - 0.71) | 0.47 |
|  | AGCAGTTC | 0.01 | 0.32 (-0.3 - 0.93) | 0.31 |
|  | ACTAGTTT | 0.01 | 0.37 (-0.42 - 1.16) | 0.36 |
| ^*^Chi–Square Test with p<0.05 is considered significant. | | | | |

| **Table S6: Post Hoc Tests for the Association of *MEF2A* and *SLC22A3-LPAL2-LPA*SNPs with variability on warfarin required doses.** | | | | | |
| --- | --- | --- | --- | --- | --- |
| **Gene** | **SNP ID** | **Genotype** | | **Initiation Dose P-value*** | **Maintenance Dose P-value*** |
| ***MEF2A*** | rs8036677 | AA | GA | 0.51 | 0.53 |
|  |  |  | GG | 0.64 | 0.63 |
|  |  | GA | AA | 0.51 | 0.53 |
|  |  |  | GG | 0.74 | 0.84 |
|  |  | GG | AA | 0.64 | 0.63 |
|  |  |  | GA | 0.74 | 0.84 |
| ***SLC22A3-LPAL2-LPA*** | rs10755578 | CC | GC | 0.45 | 0.99 |
|  |  |  | GG | 0.99 | 0.69 |
|  |  | GC | CC | 0.45 | 0.99 |
|  |  |  | GG | 0.65 | 0.73 |
|  |  | GG | CC | 0.99 | 0.69 |
|  |  |  | GC | 0.65 | 0.73 |
|  | rs2048327 | CC | CT | 0.95 | 0.79 |
|  |  |  | TT | 0.99 | 1 |
|  |  | CT | CC | 0.95 | 0.79 |
|  |  |  | TT | 0.79 | 0.40 |
|  |  | TT | CC | 0.99 | 1 |
|  |  |  | CT | 0.79 | 0.40 |
|  | rs3127599 | CC | CT | 0.63 | 0.89 |
|  |  |  | TT | 0.79 | 0.62 |
|  |  | CT | CC | 0.63 | 0.89 |
|  |  |  | TT | 0.55 | 0.51 |
|  |  | TT | CC | 0.79 | 0.62 |
|  |  |  | CT | 0.55 | 0.51 |
|  | rs366920 | CC | TC | 0.46 | 0.87 |
|  |  |  | TT | 0.35 | 0.18 |
|  |  | TC | CC | 0.46 | 0.87 |
|  |  |  | TT | 0.91 | 0.32 |
|  |  | TT | CC | 0.35 | 0.18 |
|  |  |  | TC | 0.91 | 0.32 |
|  | rs7767084 | CC | CT | 0.74 | 0.21 |
|  |  |  | TT | 0.48 | 0.33 |
|  |  | CT | CC | 0.74 | 0.21 |
|  |  |  | TT | 0.66 | 0.72 |
|  |  | TT | CC | 0.48 | 0.33 |
|  |  |  | CT | 0.66 | 0.72 |
| *Post-Hock Multiple Comparisons Test with p<0.05 is considered significant. Compare means of the initiation and maintenance dose among all genotypes.  Post hock are not performed for rs10455872, rs3125050 and rs3127583because its fewer than three groups | | | | | |

| **Table S7: The distributions of *SLC22A3-LPAL2-LPA*haplotypes among 212 warfarin responsiveness patients** | | | | |
| --- | --- | --- | --- | --- |
| **Gene** | **Haplotypes** | **Frequency (%)** | **Odds ratio (95% CI)** | **p-value^*^** |
| ***SLC22A3-LPAL2-LPA*** | ACTAGCTC | 0.29 | 0.00 | --- |
|  | ACTAGTTT | 0.1 | 0.03 (-0.16 - 0.22) | 0.77 |
|  | AGTAGCTC | 0.09 | 0.05 (-0.16 - 0.26) | 0.66 |
|  | AGTAGTTC | 0.08 | -0.05 (-0.24 - 0.14) | 0.59 |
|  | AGTAGCTT | 0.05 | -0.11 (-0.38 - 0.16) | 0.43 |
|  | ACTAGCTT | 0.05 | -0.05 (-0.39 - 0.29) | 0.77 |
|  | ACCAGTCC | 0.04 | -0.02 (-0.29 - 0.25) | 0.89 |
|  | ACCAGCTC | 0.04 | -0.08 (-0.34 - 0.18) | 0.53 |
|  | AGCAGTCC | 0.03 | 0.12 (-0.16 - 0.39) | 0.41 |
|  | ACTAGTTT | 0.03 | -0.02 (-0.36 - 0.32) | 0.91 |
|  | AGCAGTTC | 0.01 | 0.11 (-0.28 - 0.51) | 0.58 |
|  | ACTCATTC | 0.01 | -0.06 (-0.5 - 0.38) | 0.79 |
|  | AGCAGCCC | 0.01 | -0.22 (-0.68 - 0.24) | 0.35 |
|  | ACTCATTT | 0.01 | 0.04 (-0.44 - 0.52) | 0.87 |
|  | ACCAGCCT | 0.01 | -0.07 (-0.54 - 0.41) | 0.78 |
|  | AGCCATCC | 0.01 | 0.18 (-0.27 - 0.63) | 0.44 |
|  | ACCAGCTC | 0.00 | 1.13 (0.35 - 1.92) | 0.005 |
| ^*^Chi–Square Test with p<0.05 is considered significant. | | | | |

| **Table S8: Post Hoc Tests for the Association of *MEF2A* and *SLC22A3-LPAL2-LPA*SNPs with INR Treatment Outcome.** | | | | | |
| --- | --- | --- | --- | --- | --- |
| **Gene** | **SNP ID** | **Genotype** | | **Initiation INR P-value*** | **MaintenanceINR P-value*** |
| ***MEF2A*** | rs8036677 | AA | GA | 0.91 | 0.46 |
|  |  |  | GG | 0.88 | 0.71 |
|  |  | GA | AA | 0.91 | 0.46 |
|  |  |  | GG | 0.98 | 0.43 |
|  |  | GG | AA | 0.88 | 0.71 |
|  |  |  | GA | 0.98 | 0.43 |
| ***SLC22A3-LPAL2-LPA*** | rs10755578 | CC | GC | 0.38 | 0.95 |
|  |  |  | GG | 0.46 | 0.99 |
|  |  | GC | CC | 0.38 | 0.95 |
|  |  |  | GG | 0.95 | 0.99 |
|  |  | GG | CC | 0.46 | 0.99 |
|  |  |  | GC | 0.95 | 0.94 |
|  | rs2048327 | CC | CT | 0.31 | 0.99 |
|  |  |  | TT | 0.72 | 0.96 |
|  |  | CT | CC | 0.31 | 0.99 |
|  |  |  | TT | 0.39 | 0.94 |
|  |  | TT | CC | 0.72 | 0.96 |
|  |  |  | CT | 0.39 | 0.94 |
|  | rs3127599 | CC | CT | 0.99 | 0.71 |
|  |  |  | TT | 0.13 | 0.85 |
|  |  | CT | CC | 0.99 | 0.71 |
|  |  |  | TT | 0.13 | 0.98 |
|  |  | TT | CC | 0.13 | 0.85 |
|  |  |  | CT | 0.13 | 0.98 |
|  | rs366920 | CC | TC | 0.98 | 0.95 |
|  |  |  | TT | 0.39 | 0.79 |
|  |  | TC | CC | 0.98 | 0.95 |
|  |  |  | TT | 0.44 | 0.60 |
|  |  | TT | CC | 0.39 | 0.79 |
|  |  |  | TC | 0.44 | 0.60 |
|  | rs7767084 | CC | CT | 0.98 | 0.87 |
|  |  |  | TT | 0.86 | 0.83 |
|  |  | CT | CC | 0.98 | 0.87 |
|  |  |  | TT | 0.76 | 0.99 |
|  |  | TT | CC | 0.86 | 0.83 |
|  |  |  | CT | 0.76 | 0.99 |
| *Post-Hock Multiple comparisons Test with p<0.05 is considered significant. Compare initiation and maintenance dose among all genotypes.  Post hock are not performed for rs10455872, rs3125050 and rs3127583because its fewer than three groups | | | | | |
